# The non-specific lipid transfer protein from hazelnut, Cor a 8, a relevant food allergen

**DOI:** 10.1186/2045-7022-5-S3-P17

**Published:** 2015-03-30

**Authors:** Pawel Dubiela, Sabine Pfeifer, Merima Bublin, Cristine Hafner, Karin Hoffmann-Sommergruber

**Affiliations:** 1 Department of Pathophysiology and Allergy Research, Medical University of Vienna, Vienna, Austria; 2 Karl Landsteiner Institute for Dermatological Research, St. Poelten, Austria

## Background and aim

Hazelnuts are potent inducers of food allergic symptoms, ranging from mild up to severe reactions in sensitized individuals. So far, 10 hazelnut allergens were identified, among those the ns lipid transfer protein, Cor a 8. The aim of the current study is to purify and characterise the physicochemical and allergenic properties of Cor a 8.

## Methods

nCor a 8 was purified from raw hazelnuts by precipitation and chromatographical steps. Subsequently, the protein was identified by N-terminal sequencing and mass spectrometry. Stability of the protein was investigated by simulated gastric and duodenal digestion assays. Hazelnut lipids were extracted employing the hexane method. Finally, IgE binding activity of native and reduced/alkylated nCor a 8 was tested in ELISA using sera from 6 hazelnut allergic patients.

## Results

Purified nCor a 8 migrates in SDS PAGE as a single band at around 12 kDa. MALDI TOF mass spectrometry provided 9.475 kDa corresponding to the theoretical mass of 9.468 kDa (database access. number: 9QATH2). The intact n-terminus was verified by Edman-degradation verifying the first 6 amino acid residues. In digestion assays purified Cor a 8 displayed high stability against enzymatic treatment.

IgE binding activity was tested in sera from 6 hazelnut allergic patients. Three out of those sera had IgE predominantly recognizing linear epitopes as compared to the other 3 sera which had IgE specific for conformational epitopes. In RBL assays Cor a 8 induced mediator release in a dose dependent manner. Upon addition of hazelnut lipids this response was remarkably increased.

## Conclusions

Cor a 8 was purified from raw hazelnut extract, displaying the physicochemical properties of a member of the nsLTP protein family, such as high resistance against enzymatic degradation. In cellular assays the allergenic activity of Cor a 8 was considerably increased when adding hazelnut lipids, thus showing the impact of food matrix on the allergenicity of a single food protein.

